# Long-term Effects of the pituitary-adenylate cyclase-activating Polypeptide (PACAP38) in the Adult Mouse Retina: Microglial Activation and Induction of Neural Proliferation

**DOI:** 10.1007/s11064-023-03989-7

**Published:** 2023-07-19

**Authors:** Viktoria Denes, Akos Lukats, Gergely Szarka, Rovena Subicz, Adrienn Mester, Andrea Kovacs-Valasek, Peter Geck, Gergely Berta, Robert Herczeg, Etelka Postyeni, Attila Gyenesei, Robert Gabriel

**Affiliations:** 1 Department of Neurobiology, University of Pécs, 6 Ifjúság str, Pécs, H-7624 Hungary; 2 Institute of Translational Medicine, Translational Retina Research Group, Semmelweis University, Budapest, Hungary; 3 Department of Medical Education, School of Medicine, Tufts University, 136 Harrison Ave, Boston, MA 02111 USA; 4 Department of Medical Biology and Central Electron Microscope Laboratory, Medical School, University of Pécs, Pécs, Hungary; 5 János Szentágothai Research Centre, Bioinformatics Research Group, University of Pécs, 20 Ifjúság str, Pécs, H-7624 Hungary

**Keywords:** PACAP38, Adult retina, Microglia, Inflammation, Angiogenesis, Proliferation

## Abstract

The degenerative retinal disorders characterized by progressive cell death and exacerbating inflammation lead ultimately to blindness. The ubiquitous neuropeptide, PACAP38 is a promising therapeutic agent as its proliferative potential and suppressive effect on microglia might enable cell replacement and attenuate inflammation, respectively. Our previous finding that PACAP38 caused a marked increase of the amacrine cells in the adult (1-year-old) mouse retina, served as a rationale of the current study. We aimed to determine the proliferating elements and the inflammatory status of the PACAP38-treated retina. Three months old mice were intravitreally injected with 100 pmol PACAP38 at 3 months intervals (3X). Retinas of 1-year-old animals were dissected and effects on cell proliferation, and expression of inflammatory regulators were analyzed. Interestingly, both mitogenic and anti-mitogenic actions were detected after PACAP38-treatment. Further analysis of the mitogenic effect revealed that proliferating cells include microglia, endothelial cells, and neurons of the ganglion cell layer but not amacrine cells. Furthermore, PACAP38 stimulated retinal microglia to polarize dominantly into M2-phenotype but also might cause subsequent angiogenesis. According to our results, PACAP38 might dampen pro-inflammatory responses and help tissue repair by reprogramming microglia into an M2 phenotype, nonetheless, with angiogenesis as a warning side effect.

## Introduction

To sense the complex visual world surrounding us (i.e. colors, shapes, orientations, movements), a similarly complex system evolved that includes the highly organized neural circuitries of the retina. Herein, a narrow spectrum of electromagnetic waves is not only converted into electrical signals but partially processed and ultimately transmitted toward the visual centers of the brain. Obviously, retinal diseases causing blindness or impaired vision, which can affect people of all ages pose a great burden on individuals and society in general. Therefore, seeking therapeutic agents to cure, prevent, or just alter the severity of retinal disorders is one of the leading aims of the retinal research community.

The degenerative retinal disorders (e.g. age-related macular degeneration, diabetic retinopathy, retinitis pigmentosa, retinal detachment) are characterized by progressive and irreversible loss of retinal cells including photoreceptors, pigmented epithelial cells, or retinal ganglion cells. Pathogenesis of these conditions is often exacerbated by inflammation mediated and performed by residential macrophages, and retinal microglia cells [1]. Thus, demand is growing for therapeutical agents enabling cellular replacement and/or affecting the microglial system.

One of the promising therapeutic agents is the pituitary-adenylate cyclase-activating polypeptide (PACAP38) which represents an actively studied field in retinal research, bioactive neuropeptides. This highly conserved bioactive molecule exists in shorter (27 aa) and longer (38 aa) isoforms in a wide range of living organisms from invertebrates to vertebrate species [2]. PACAP38 has the strongest bias toward the PAC1-receptor but also signals via VPAC1 and VPAC2 receptors sharing the affinity with a close family member, the vasoactive intestinal peptide (VIP) [3]. In addition, undergoing an extensive splicing, N-terminal, as well as intracellular loop variations of PAC1-receptor are generated with various affinity and, more importantly, various intracellular signaling. All PACAP receptors are metabolic receptors coupled to diverse downstream transduction pathways including the activation or inhibition of adenylate cyclase, activation of phospholipase C, and/or phospholipase D. Due to its ubiquitous expression and the outstanding diversity of the receptor signaling, PACAP38 has been reported to possess pivotal roles in numerous physiological processes [4, 5]. Extensively studied, a large body of data has been accumulated proving the advantageous as well as the adverse effects of this multi-facetted peptide in a disparate array of pathological conditions [6]. Accordingly, the beneficial effects of PACAP38 were studied not only in the aspects of retinal degenerative models (i.e. ischemia, diabetic retinopathy, glutamate, -and UVB-induced cell death) [7] but in the natural processes of retinal development and aging [6, 8]. The transcription of PAC1-receptor gene was revealed in adult rat RGCs and amacrine cells by in situ hybridization technique [9] and PAC1-receptor positive RGCs, horizontal cells, amacrine cells, and Muller glial cells were observed in the developing retina from P3 through P10 [6, 10, 11]. While the expression of PAC1 receptor is well-documented in rat retina, available morphological data are rather spare in the mouse retina.

Regulation of cell division is one of the most studied functions of PACAP38 and it could shed light on a new avenue for progenitor-mediated regeneration and tissue repair. Nevertheless, PACAP38 appeared to be Janus-faced inducing both proliferation [12–14] and cell cycle arrest [15, 16] in a tissue-, concentration- or receptor-dependent manner [17, 18]. Our research group also found marked horizontal cell proliferation at postnatal day 3 (P3) or 8 (P8) induced by intravitreal PACAP38 injection in rat retina [6]. Furthermore, PACAP38 also caused significant cell number elevations of the somatostatinergic and dopaminergic cells in 12,- and 18 months old murine retina, when repeated intravitreal injections were performed to investigate the effect of PACAP38 on retinal aging [8]. These findings served as the basis of the current study, which addressed the mechanism underlying the above effect of PACAP38 on the amacrine cell populations. One-year-old retinas were investigated, which allowed us to observe the cumulative effects of three intravitreal injections. We proposed that PACAP38 could induce the re-entry of somatostatinergic and dopaminergic amacrine cells into the cell cycle. The data we present here, however, did not support our initial hypothesis. Although PACAP38 exerted both mitogenic and anti-mitogenic effects in the adult mouse retina, did not induce the cell cycle in somatostatinergic or dopaminergic amacrine cells. Unexpectedly, further histology studies revealed that the proliferating elements of the PACAP38-treated retina were a certain set of neurons in the ganglion cell layer (GCL), microglial cells, and endothelial cells. We report here, for the first time, that PACAP38 exerts an advantageous impact on the retinal microglia population. Furthermore, we show that the inflammatory status of the PACAP38-treated retina seems to promote M2 polarization of retinal microglia where the dominant activity is tissue recovery and tissue support. On the other hand, caveat emptor: our findings also represent a warning for potential angiogenesis as a side effect of PACAP38 in the retina. The results warrant further studies to dissect and understand the puzzling effects of this powerful and potentially therapeutic neuropeptide.

## Experimental Procedure

### Experimental Animals

For all investigations, C57Bl/6JTdTomato transgenic mice were purchased from the Institute of Experimental Medicine, Hungarian Academy of Sciences. Mice were generated by crossing homozygote SST/iresFlpo (Tm3) mouse line with homozygote GT(ROSA)26Sor_CAG/FSF TdTomato mice. Accordingly, all somatostatin-expressing cells exhibit an enhanced red fluorescent protein (TdTomato) in these animals. Validation of the construct was performed previously by Pöstyéni et al. [8]. Animals were housed in a temperature and light-controlled room (12/12 h light/dark cycles, 23 °C), provided with *ad libitum* water and food supply.

### Treatments and Dissection

All experimental procedures were conducted according to the guidelines of the Declaration of Helsinki, and approved by the Animal Health and Animal Welfare Directorate of the National Food Chain Safety Office of the Hungarian State (PE/EA/488-6/2021), and the European Animal Research Association. All efforts were made to prevent pain and suffering. Animals were anesthetized in 2–3% isoflurane (Abbott Laboratories, Budapest, Hungary) prior to the treatments. The intravitreal injections were performed at the age of 3, 6, and 9 months. In the right eyes, 1.5 µl 0.3 µg/µl (100 pmol) PACAP38 (Bio Basic Canada Inc., Markham, Ontario, Canada) was injected with 33G needles attached to a 10 µl microsyringe to the depth of 1 mm. The paired eye served as a vehicle control receiving the same volume of sterile 0.9% saline solution. Three months after the last injection, the 1-year-old mice were euthanized by inhalation of 3% isoflurane and their eyes were removed. Furthermore, an additional group of age-matched animals that received absolutely no intravitreal injections were also formed and will be referred to as untreated control throughout the text. The PACAP38-treated, saline-treated and untreated retinas were further processed for downstream applications as described below.

### Immunohistochemistry on Cryosections

For immunohistochemistry, eyes were enucleated and eyecups were prepared. Using a 21G needle, the anterior chamber was opened and then micro scissor was used to create a circular incision 1 mm posterior to the limbus. This resulted in the removal of the entire cornea and iris. Lens and vitreous body were gently removed with a fine tweezer. The eyecups were fixed in 4% phosphate-buffered paraformaldehyde for 2 h at room temperature followed by rinsing in phosphate-buffered saline (PBS) for 3 × 10 min. To prevent crystal formation, the eyecups were sequentially immersed into 10-20-30% sucrose solution and then embedded in a tissue embedding medium (Shandon Cryomatrix, ThermoFisher Scientific, Budapest, Hungary). 10–15 μm thick sections were cut in a cryotome and stored at -20 °C. Prior to incubation in primary antibodies, sections were permeabilized in 0.3% Triton X-100 in PBS (Merck, Budapest, Hungary) for 30 min. In the case of PCNA immunolabelling, enhanced permeabilization was required thus incubation in ice-cold 99.95% methanol was added for 10 min at -20 °C. To block non-specific binding sites, sections were incubated in 5% normal serum and 1% bovine serum albumin dissolved in PBS containing 0.3% Triton X-100 for 30 min. All primary antibodies were applied overnight at room temperature. Detailed information on the primary antibodies is provided in Table 1. The bound primary antibodies were visualized by fluorochrome-conjugated secondary antibodies (Table 2). Blood vessels were labeled with Alexa Fluor 647 Isolectin GS-IB4 conjugate (1:500, ThermoFisher Scientific, Budapest, Hungary) incubated simultaneously with the secondary antibodies. Finally, the sections were mounted with ProLong Gold antifade reagent with 4’-6-diamidino-2-phenylindole (DAPI) to counterstain the nuclei (Life Technologies, Budapest, Hungary). As negative controls, primary antibodies were omitted in both single- and double-labeling experiments, which resulted in no signal. Cross-reactivity of the non-corresponding primary and secondary antibodies was also checked. Immunostainings were examined on tissues derived from two or three different animals. In order to investigate identical, central parts of both control and treated retinas, the optic discs were used as reference points.

**Table 1:** List of primary antibodies applied for immunohistochemistry

Name	Host and type	Dilution	Supplier/ Cat. number
anti-PAC1 receptor	rabbit polyclonal	1:500	ThermoFisher Scientific, Budapest Hungary (PA3-115)
anti-proliferating cell nuclear antigen (PCNA)	rabbit polyclonal	1:1000	Cell Signaling Technology, Danvers, Massachusetts, USA (3110)
anti-ionized calcium binding adaptor molecule-1 (Iba-1)	guinea pig monoclonal	1:2000	Synaptic Systems, Göttingen, Germany (234 308)
anti-VPAC1 receptor	mouse monoclonal	1:100	GeneTex, Irvine, California, USA (GTX16155)
anti-VPAC2 receptor	mouse monoclonal	1:100	ThermoFisher Scientific Budapest Hungary (MA1-12904)
anti-tyrosine-hydroxylase (TH)	mouse monoclonal	1:1000	Merck, Budapest Hungary (T2928)
anti-gluthamine synthetase	rabbit polyclonal	1:1000	Merck, Budapest Hungary (G2781)

**Table 2:** List of secondary antibodies applied for immunohistochemistry

Name	Fluorochrome	Dilution	Supplier
anti-rabbit IgG	Alexa 448	1:500	ThermoFisher Scientific, Budapest Hungary
anti-rabbit IgG	Alexa 647	1:500	ThermoFisher Scientific, Budapest Hungary
anti-guinea pig IgG	Alexa 647	1:500	ThermoFisher Scientific, Budapest Hungary
anti-mouse IgG	Alexa 488	1:500	ThermoFisher Scientific, Budapest Hungary

### Confocal Imaging

Images were taken either by a Zeiss LSM 710 confocal laser scanning microscope (Carl Zeiss Inc., Jena, Germany) or an Olympus FV-1000 laser scanning confocal fluorescence microscope (Olympus, Hamburg, Germany).

### Cell Counting

Microglia cell counting was performed on complete, 10–12 μm thick retinal sections derived from 1 untreated control, 2 vehicle control, and 3 PACAP38-treated retinas. Every fourth section was selected from the serial sections to avoid repeated counts of the same cells; a minimum of 3 sections per specimen were used, which included the optic nerve head. The full thickness of the selected Iba-1/DAPI double-labeled retinal sections was scanned by a Zeiss LSM 710 confocal laser scanning microscope. The tile scans were imported without any alterations to FIJI/Image-J [19] and maximum intensity projections were created. Only microglia cells, with the nucleus in the plane of the section, were counted and the results were expressed as microglia cells/section ± standard deviation (SD). The number of samples (n) refers to the number of sections analyzed in the experimental groups (i.e. untreated control, vehicle control, PACAP38-treated).

### Measurement of Microglial area Fraction

To estimate the area occupied by microglia somata and processes, the same tile scans were used as described above. The brightness of the Iba-1 labeling was adjusted so that randomly selected non-labeled background tissue was at 0% and at least two randomly selected Iba-1 positive cells somata were at 100% intensity. Selecting the Default threshold method, a binary mask was created from the Iba-1 labeling. The total retinal area was selected based on the DAPI signal including the whole section from the GCL to RPE using the selection brush tool and stored as a region of interest (ROI), then the Iba-1 area fraction was determined utilizing the measure tool.

### Quantitative real-time PCR (Q-PCR)

For molecular application, retinas were dissected in RNase-free, cold PBS. Whole retinal tissues were frozen on dry ice upon removal and stored at -80 C° until extraction.

Total RNA was extracted using QIAshredder and RNeasy Plus Mini Kit (Qiagen, Germantown, Maryland, USA) in accordance with the manufacturer’s instructions. The quality of the RNA samples was determined by measuring optical density at 260 nm with BioPhotometer Plus (Eppendorf, Hamburg, Germany). Purity was estimated by the 260/280 nm absorption ratio, which was consistently higher than 1.8. Reverse transcription was carried out from 500 ng up to 1 µg of total RNA to convert RNA into first-strand cDNA. The cDNAs were run in Mouse Cell Cycle RT² Profiler™ PCR Arrays (Qiagen, Germantown, Maryland, USA) following the manufacturer’s instructions. In these arrays, the cDNAs of three vehicle control samples were pooled and used as a reference. Furthermore, three PACAP38-treated retina samples were run providing three biological replicates. The ΔΔCt method was used to calculate relative fold-change values, which were log2 transformed and referred to as fold-regulation. To assess the predominant microglial phenotype in the PACAP-treated tissues, custom primers were designed to probe arginase-1 (Arg-1), inducible nitric oxide synthase (iNOS), CD16, and CD14 mRNAs. Four pairs of vehicle-control and PACAP38-treated retinas were compared to a pooled untreated control sample, which served as a basis for relative quantitation. The expression differences were determined between each pair and averaged. To normalize signals, two constitutively expressed, endogenous controls were utilized: ribosomal protein L13a (RPL13a) and lactate dehydrogenase (LacD). Primer sequences are shown in Table 3. Expression changes were calculated with ΔΔCt method and adjusted according to the efficiency of each primer pair. Q-PCR data represent the means of 4 biological replicates and are expressed as fold changes ± SD.

**Table 3:** Primer sequences used in Q-PCR runs

Name	Forward Primer (5’-3’)	Reverse Primer (5’-3’)
Arg-1	TAC CTT AAA CCA CCT AAG T	TTT ATA GCT GTT TTG ATT TCC
iNOS	GAA GAA ATG CAG GAG ATG	GCT TCT TCA ATG TGG TAG
**CD16**	**AAA CTA CTG AAC AGG ATC T**	**AGA TGG AGG ATG TAG TTG**
**CD14**	**CTA GAC CTT AGT CAC AAT TC**	**CCT TTA AGT GAC AGG TTC**
**RPL13a**	**TCT GAG CAT CTC TTT CTC TCA AC**	**CTT CTT CTT CCG ATA GTG CAT CT**
**LacD**	**CAG AAG GAG CTG CAG TTC TAA AG**	**CTA ACC AGG TCA CCA CTA CAC AA**

### nCounter Neuroinflammation Panel

The nCounter technology (NanoString Technologies, Seattle, Washington, USA) is a new tool that uses molecular bar codes enabling direct detection and counting of native RNA transcripts thus determining gene expression without amplification. Preparation, hybridization, and loading were performed following the manufacturer’s manual. The integrity of total RNA samples was verified via Agilent Bioanalyzer 2100 instrument (Agilent Technologies, Santa Clara, California, USA) using Agilent RNA 6000 Nano reagent kit with Eukaryotic Total RNA assay. Fragment analysis and the degree of degradation were determined before proceeding. The percent of the fragments larger than 200 nt was estimated and turned out to be higher than 90% in every sample. Equal amounts of total RNAs from three non-treated, and five saline-treated retinas were pooled. In addition to the pooled untreated and vehicle controls, five PACAP-treated samples were examined using Mouse nCounter Neuroinflammation Panels (NanoString Technologies, Seattle, Washington, USA). The hybridization reactions were composed of a hybridization buffer, reporter code set, 30 ng RNA sample, and capture probe set in a total volume of 15 µl. Hybridization reactions were incubated at 65 °C for 19 h. Reactions were brought to 35 µl with RNAse-free water and loaded immediately into the cartridge and proceeded in the nCounter SPRINT Profiler instrument (NanoString Technologies, Seattle, Washington, USA).

### Data Analysis

To analyze the data collected in reporter library file (RLF) and reporter code counts (RCC), the nSolver Advanced Analysis Software v4.0.70 (NanoString Technologies, Seattle, Washington, USA) was utilized. Necessary adjustments (i.e. background correction, quality assessment, and inter-sample normalization) were performed following the Gene Expression Data Analysis Guidelines (MAN-C0011-04). Lanes with a registered Field of View (FOV) appeared under 75% were flagged. Binding densities were accepted if the optical feature per square micron fell into the range of 0.1 to 1.8. The performance of the positive controls was assessed by checking positive control linearity and positive control limit of detection; set to correlation of 0.95 and ≤ 2 SD above the mean of the negative controls, respectively. The mean of the negative controls plus two standard deviations was used to set Background Thresholds. For positive control normalization, geometric means of the positive controls were taken to calculate normalization factors for each sample (range of 0.3-3.0), while the geometric mean of the reference genes were used to compute normalization factors (range of 0.1–10.0) for Codeset content normalization. Significance was assigned for differentially expressed genes with a p-value of ≤ 0.05.

Regarding the analyses of microglial cell count and area fraction measurement, following a test of normality, the statistical comparisons were performed by one-way ANOVA analysis. Experimental groups were pitted against each other using the posthoc Tukey test. A value of p ≤ 0.05 was considered statistically significant.

## Results

### Both Dopaminergic and Somatostatinergic Cells of the Adult Mouse Retina Express PAC1-receptor

**Fig. 1:**
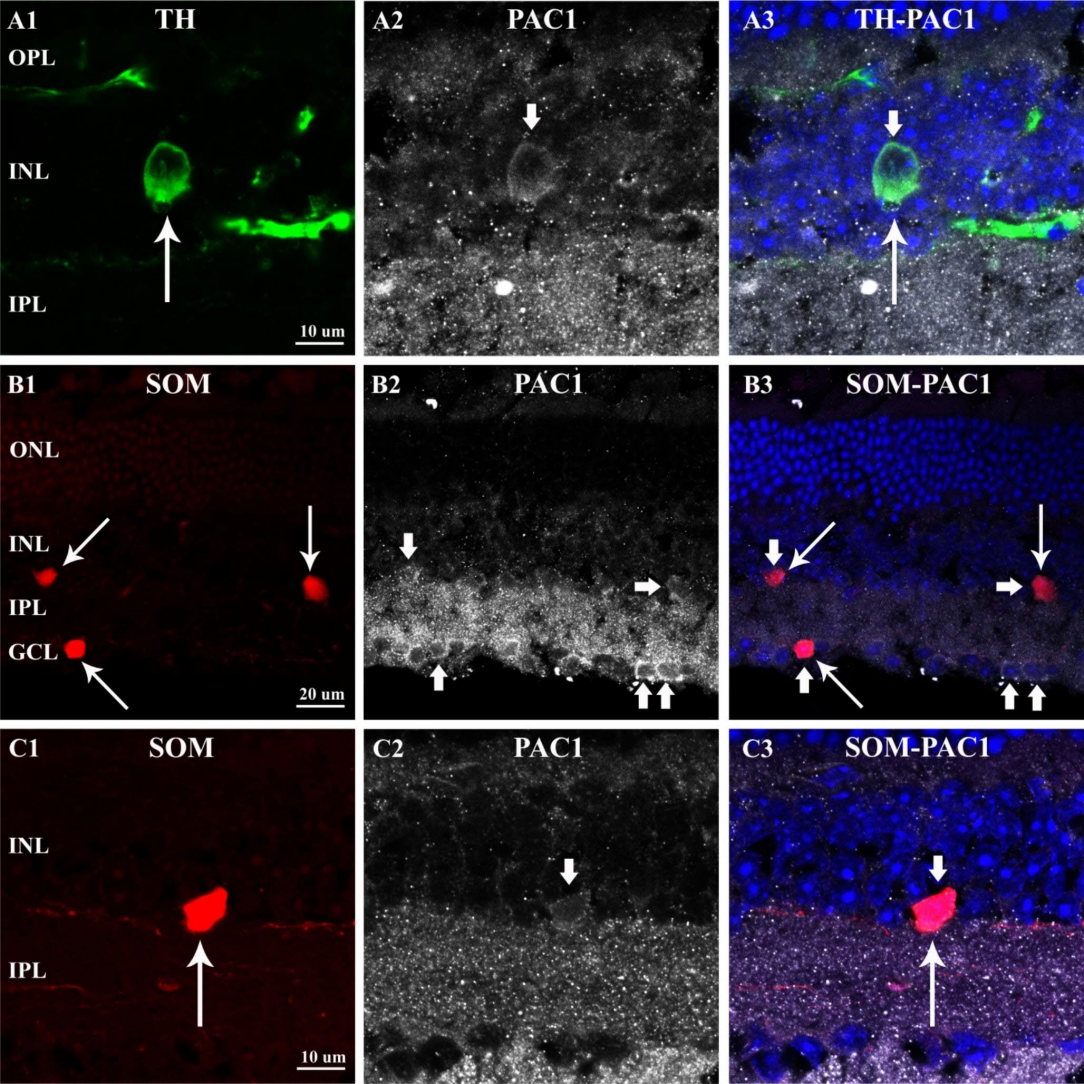
Confocal fluorescence micrographs of PAC1-R expressing TH-IR and somatostatinergic amacrine cells of the 1-year-old mouse retina. In the (A1) panel, TH-IR dopaminergic neuron is revealed (thin arrow). (A2) PAC1-IR immunostaining and merge (A3) of the two stainings prove the co-expression of TH and PAC1-receptor. (B1) Somatostatinergic amacrine cells located in the INL and in the GCL (thin arrows). (B2) PAC1-IR cell population is labeled in the INL and GCL (thick arrows). (C1) shows a tdTomato-expressing somatostatinergic amacrine cell at higher magnification, which is also immunoreactive for PAC1-receptor shown in C2. Co-labeling is presented in C3 (ONL-outer nuclear layer; OPL-outer plexiform layer; INL-inner nuclear layer, IPL-inner plexiform layer; GCL-ganglion cell layer)

First, we investigated whether the somatostatinergic or dopaminergic cells expressed PAC1-receptor, which might reflect a direct effect on their population. In Fig. 1, tyrosine-hydroxylase (TH) and PAC1 co-labeling is shown. TH-immunoreactive (-IR) neuron (A1, thin arrow) was double-labeled with PAC1-receptor (A2, thick arrow) as depicted clearly in the merged pictures (A3). Likewise, somatostatinergic (red, tdTomato-expressing) cells (B1, thin arrows) both in INL and GCL appeared to be PAC1-IR (B2, thick arrows). Notably, numerous PAC1-IR cells were seen in the GCL (B2 and B3, thick arrows). In Fig. 1C1-C3, a double-labeled somatostatinergic amacrine cell is shown at higher magnification.

#### Multiple PACAP38 Injections Induce Proliferation as well as cell Cycle Arrest

In our previous study, PACAP38 was demonstrated to induce horizontal cells to re-enter the cell cycle via PAC1-receptor in the postnatal rat retina (i.e. P8) [6]. This finding served as a rationale to examine the proliferation state of the PACAP38-treated retina. Three PACAP38-treated retinas were processed and run in Cell Cycle RT² Profiler™ PCR Arrays composed of genes that are considered to serve as pro- and anti-proliferative drivers. Fold-regulation values greater than 2 or lower than − 1.5 are presented in separate columns in Tables 4 and 5. Separate columns represent results obtained from different samples thus showing the possible variations within. Instances, where no change was detected, are demarcated by N/Ch. Table 4 summarizes the upregulated genes that are involved in mitosis (10) and the downregulated anti-mitotic factors (6). The expression changes of these genes suggested an increased proliferation of certain retinal elements in the treated retinas. The results indicate, however, that PACAP38 also had the opposite effect on cell division causing cell cycle arrest by upregulation of anti-mitotic factors (6) and suppressing quite a few mitotic factors (6) listed in Table 5.

**Table 4:** Factors indicating the proliferative effect of PACAP in 1-year-old mouse retinas (n = 3). Values show the fold-regulation. N/Ch abbreviation stands for not changed

Mitotic factors UPregulated				Anti-mitotic factors DOWNregulated			
Genes	Fold regulation			Genes	Fold regulation		
**Abl1**	4.37	4.13	3.34	**Cdkn1b**	N/Ch	-2.94	-1.76
**Ccnb1**	4.61	2.77	13.25	**E2f4**	-2.29	-2.55	N/Ch
**Mcm3**	2.89	2.00	2.52	**Mad2l1**	N/Ch	-3.49	-1.5
**Nek2**	3.57	2.57	7.45	**Rbl2**	-1.77	-4.23	N/Ch
**Aurka**	3.08	N/Ch	4.09	**Tfdp1**	-1.55	-2.67	N/Ch
**Aurkb**	57.72	N/Ch	72.97	**Rad17**	N/Ch	-2.7	-1.5
**Birc5**	3.23	N/Ch	3.01				
**E2f2**	55.57	N/Ch	22.56				
**Cdc25a**	N/Ch	2.00	2.70				
**Cdk1**	N/Ch	2.35	2.7				

**Table 5:** Factors involved in the anti-proliferative effect of PACAP in 1-year-old mouse retinas (n = 3). Values show fold-regulation. N/Ch abbreviation stands for not changed

Anti-mitotic factors UPregulated				Mitotic factors DOWNregulated			
Genes	Fold regulation			Genes	Fold regulation		
**Gpr132**	5.30	2.90	5.07	**Rad51**	-3.41	-5.89	-2.02
**Slfn1**	2.17	2.87	6.85	**Sfn**	-13.86	-14.81	-4.83
**Cdkn2a**	51.95	N/Ch	119.45	**Cdc7**	N/Ch	-3.58	-1.84
**Chek2**	51.62	N/Ch	109.37	**Rad21**	-1.56	-3.00	N/Ch
**Cdkn1a**	2.03	N/Ch	2.45	**Ccna2**	N/Ch	-3.06	-1.53
**Check1**	2.00	N/Ch	2.96	**Cdc7**	N/Ch	-3.58	-1.84

### PCNA-IR Neural Elements of the Adult Mouse Retina After Multiple PACAP38 Injections

**Fig. 2:**
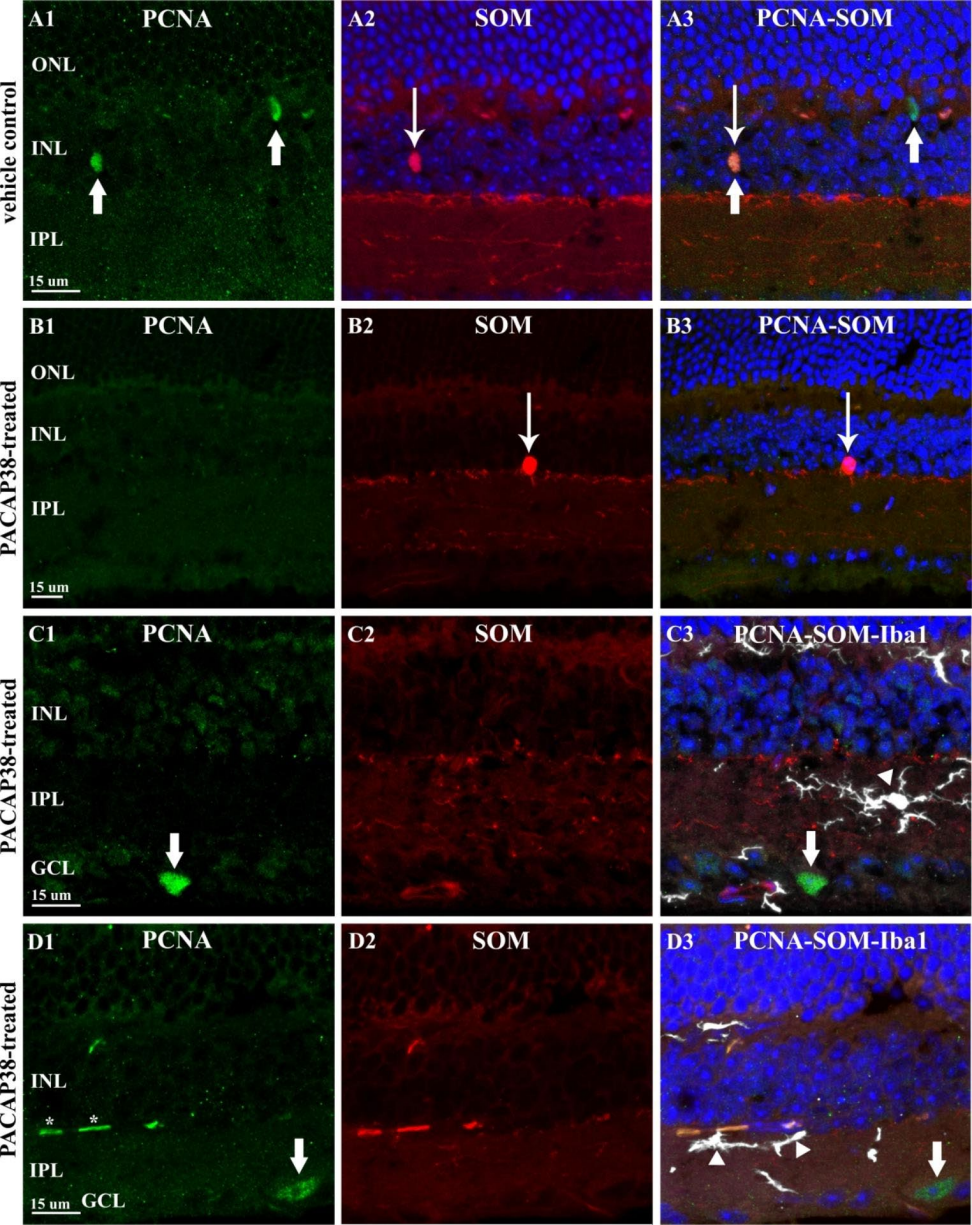
Identification of PCNA-IR neural elements in the 1-year-old mouse retina. (A1) Thick arrows point to PCNA-IR nuclei seen in the INL and the OPL of the vehicle control retina. In (A2) picture, a single tdTomato-expressing cell (thin arrow) is seen in the INL, which is identified as a nucleus of a somatostatinergic amacrine cell (A3). The other cell nucleus in the OPL is identified as microglia presented in Fig. 3. (B1-B3) In the PACAP38-treated retinal section, PCNA-IR (B1) somatostatinergic amacrine cells (B2, thick arrow) were not found. C1-C3 and D1-D3 depict examples of PCNA-IR cells (C1, C2, thick arrows) observed in the GCL of PACAP38-treated retinal sections. The PCNA-IR nuclei (thick arrows in C1 and D1) lacked tdTomato signal (C2, D2) and were immunonegative for Iba-1 (arrowheads in C3, D3). Note the asterisks labeling likely endothelial nuclei in D1 (ONL-outer nuclear layer; OPL-outer plexiform layer; INL-inner nuclear layer, IPL-inner plexiform layer; GCL-ganglion cell layer)

**Fig. 3:**
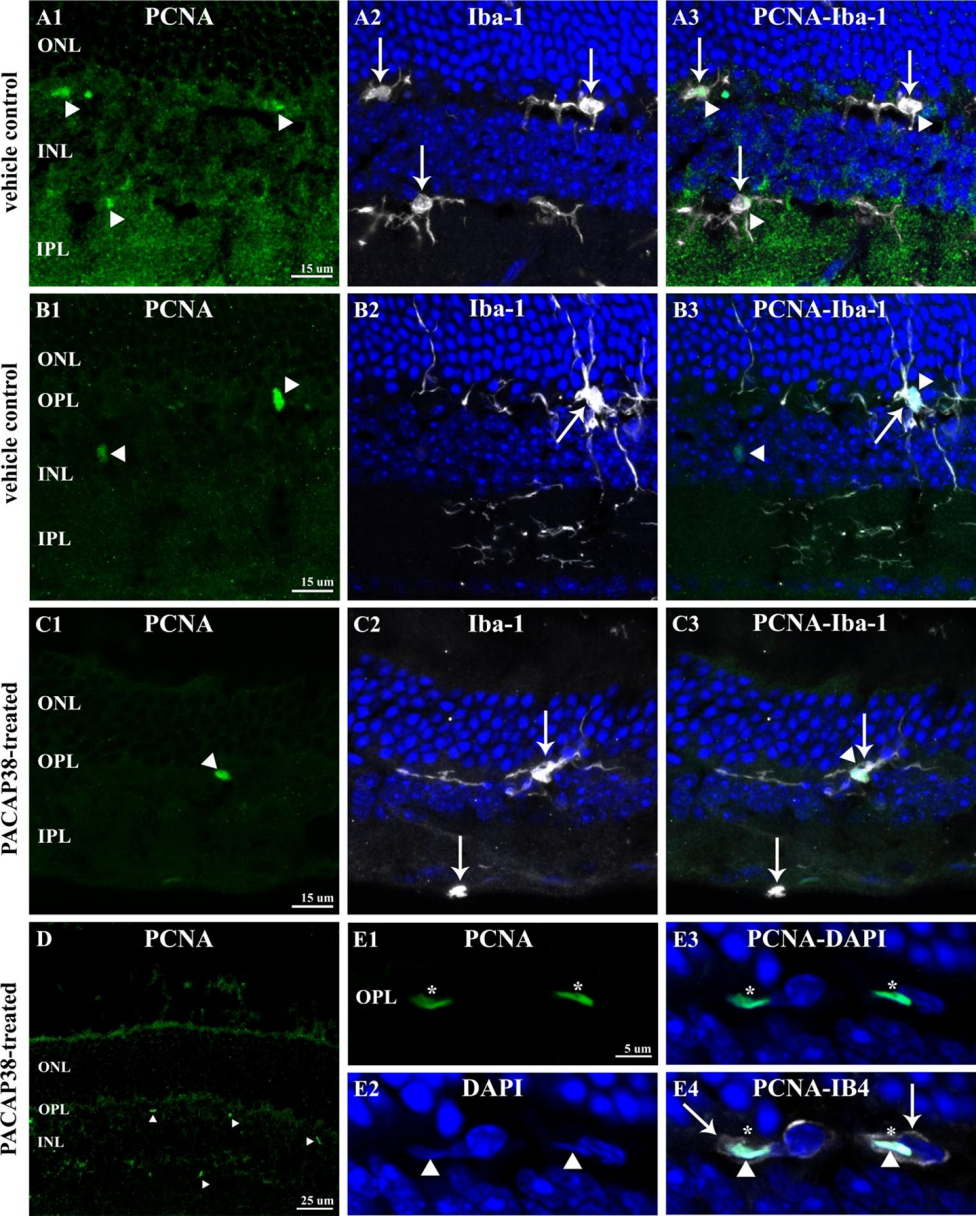
PCNA-IR non-neural structures of the 1-year-old mouse retina. (A1-A3) Three PCNA-immunopositive nuclei (A1, arrowheads) are depicted in the OPL and IPL. All three co-localized with Iba-1 (A2, arrows) in the control retina. (B1-B3) Another example of PCNA-IR cells (B1, arrowheads) in the control retina. The one in the OPL is Iba-1-IR (B2, arrow) whereas the cell seen in the INL lacks Iba-1 immunoreactivity. (C1-C3) In the OPL of the PACAP38-treated retina, a PCNA-IR nucleus is demonstrated (C1, arrowhead), which co-localized with Iba-1 (C2, arrow). In C2, an example of Iba-1-positive but PCNA-negative microglia is also shown. (D) Arrowheads label numerous, small PCNA-IR nuclei in the PACAP38-treated retina. (E1-E4) The elongated PCNA-IR structures (E1, asterisks) appear to be nuclei (E2, arrowheads) belong to isolectin-labeled endothelial cells (E4, arrows) (ONL-outer nuclear layer; OPL-outer plexiform layer; INL-inner nuclear layer, IPL-inner plexiform layer; GCL-ganglion cell layer)

In order to specify, which retinal elements were promoted to proliferate by long-term PACAP38 treatment we performed proliferating cell nuclear antigen (PCNA) immunolabeling. We found no dopaminergic amacrine cells that appeared to be mitotic as TH-PCNA co-labeling did not reveal any co-localization between the two immunostainings (not shown). In contrast, somatostatinergic neurons Fig. 2A1-A3 demonstrate PCNA-positivity in the 1-year-old vehicle control retinas. Figure 2A1-A2 depicts two PCNA-IR cells (thin arrow) and the TdTomato-expressing elements (fibers and a neuron) (thick arrow) of the control retina, respectively. The merged image reveals (A3) that the PCNA-IR cell seen in the INL is a somatostatinergic amacrine cell. The other PCNA-IR cell found in the OPL is microglia, the same cell is shown in a better plane in Fig. 3B1-B3. Surprisingly, no PCNA-IR cell was observed amongst the somatostatinergic neuron population in the PACAP38-treated retinas (Fig. 2B1-B3). Instead, quite a few cells undergoing mitosis were mapped in the GCL of the PACAP38-treated tissues, which could not be found in the control retinas. Two examples of such PCNA-IR cells are shown in Fig. 2C1 and D1 (thick arrow). These cells did not express tdTomato as demonstrated in C2 and D2 thus appear not to be somatostatinergic. In neural tissues, proliferating microglial cells frequently reside among the neurons thus immunostaining with microglial Iba-1 marker was performed as Iba-1 is highly specific for macrophages [20] thus widely used for microglia detection throughout the nervous system. In panels C3 and D3 (arrowheads), we show that PCNA-IR cells of the GCL lacked Iba-1 immunopositivity suggesting that these cells are neurons induced to divide.

#### PCNA-IR non-neural Elements of the Adult Mouse Retina After Multiple PACAP38 Injections

PCNA-IR nuclei were also observed in the neuropil layers, in the OPL and IPL, especially in the saline-treated vehicle controls (Fig. 3A-C, arrowheads). Due to their location and size, these nuclei could potentially belong to microglia cells thus in order to specify we performed combined PCNA and Iba-1 immunostainings. Micrographs of A1-A2-A3 show a vehicle control section with three PCNA/Iba-1 double-labeled cells. Fig. 3B1-B3 is identical to Fig. 2A1-A3, however, focusing on the PCNA-IR cell in the OPL (A1), which was co-labeled with Iba1 (A2). Proliferating microglial cell was seen in a company with PCNA-immunonegative microglia in the PACAP38-treated retinal section demonstrated in Fig. 3C1-C3.

**Fig. 4:**
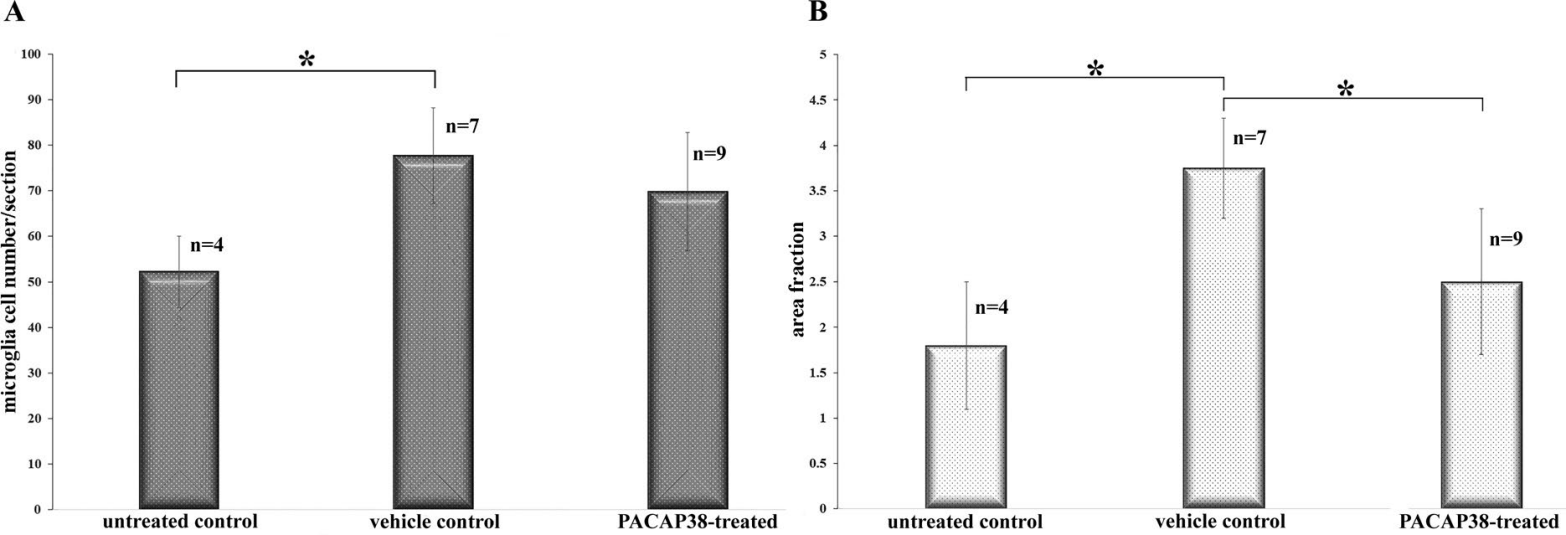
Summary of microglia cell counting (A) and area fraction measurement (B). (A) Diagrams depict the averaged microglia cell number/section ± SD in untreated, saline-treated and PACAP38-treated retinas. As a consequence of the injection, microglial cell number increased significantly in the vehicle-control and stayed elevated in the PACAP38-treated retinas as well. (B) Area fraction results show that microglia occupied significantly larger area ± SD in the vehicle control compared to the untreated control, then, the area was significantly reduced by PACAP38. Mean values ± SD of retinal cross-sections are given. Statistical comparisons were performed by one-way ANOVA analysis followed by posthoc Tukey test. Asterisks indicate p-values ≤ 0.05

Based on the visual observations, further investigations were undertaken on microglial cells determining their total number (Fig. 4A), and area fraction (Fig. 4B) taken up from a whole retinal section. Interestingly, we found a statistically significant elevation of the microglia number in the saline-treated retinas (77.71 ± 10.46; n = 7) as compared to the untreated control (52.25 ± 7.8; n = 4), which was presumably caused by the intravitreal injections (Fig. 4A). It is remarkable that the change was detectable even 3 months after the last treatment. In the PACAP38-treated tissues (69.77 ± 13; n = 9), the microglia number did not differ significantly from the vehicle control. The area fraction measurements were carried out in the same sections. In comparison to the untreated control (1.8 ± 0.7; n = 4), microglia occupied a significantly larger area in the vehicle control (3.75 ± 0.55; n = 7) corresponding to their increased number (Fig. 4B). In the case of PACAP38 treatment, while their numbers were not significantly lower, microglial cells covered a significantly smaller area in the PACAP38 injected tissues (2.5 ± 0.8; n = 9) compared with the vehicle control.

Besides the microglial nuclei, PCNA immunoreactivity was also seen in numerous, small structures (arrowheads in Fig. 3D, and asterisks in Fig. 2D1) in the PACAP38-injected retinal sections. It is clearly visible at higher magnification that the PCNA signal (Fig. 3E1) overlaps with DAPI (Fig. 3E2, E3), moreover, the isolectin GS-4 labeling seen in the merged picture (E4) proves that the PCNA-IR structures are nuclei of endothelial cells.

### Multiple PACAP38 Injections lead to Microglia Activation and Angiogenesis

**Fig. 5:**
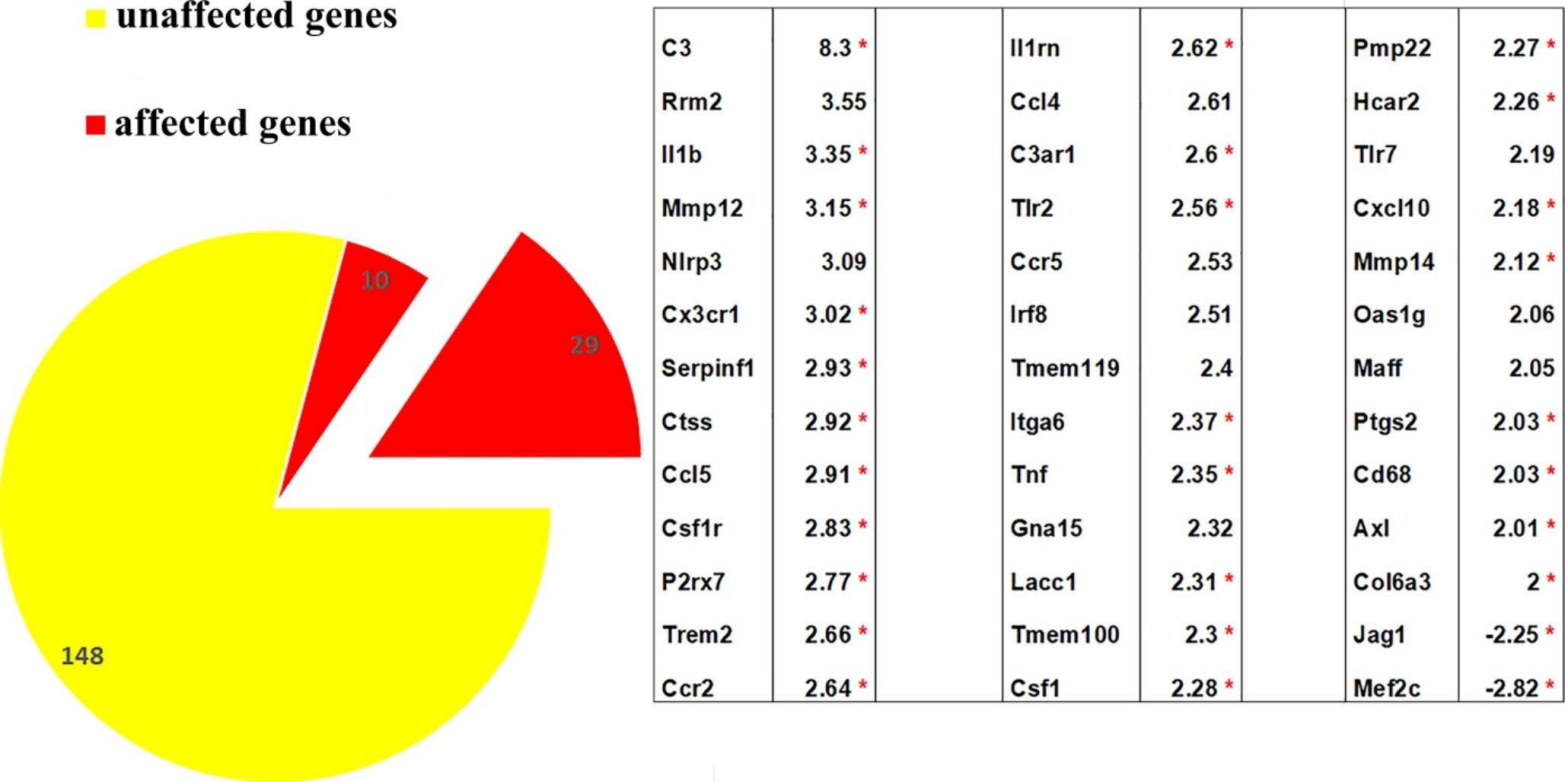
Summary of gene expression results linked to microglia function from nCounter Neuroinflammation panel. Fold-regulation values are arranged from largest to smallest. Statistically significant changes (p ≤ 0.05) are marked with red asterisks

Although the number of the microglia seemed to be unchanged, the significantly reduced area occupied by the microglias might be the consequence of changes regarding the length and/or the number and/or ramification of microglial processes. It is well-established that any of these changes and their combinations could be the signs of their activation [21]. Therefore, nCounter Neuroinflammation panel was utilized, which provided a comprehensive evaluation of the pathways involved in neuroimmune interactions including 187 genes related to microglia function. In the current study, only the aspects of microglia activation and angiogenesis are analyzed and discussed. Figure 5 presents the fold-regulation results of microglia activation obtained from five PACAP38-injected retinas. 39 out of 187 genes that displayed larger than 2-fold or smaller than − 2-fold change, 29 out of 39 appeared to be significantly different.

The message level of most of the genes was changed moderately (i.e. 2 up to 3.55-fold increase) with the exception of the factor of complement 3 (C3), which displayed an 8.3-fold upregulation. Only two genes appeared to be down-regulated; Jag1 and Mef2c.

Although the PCNA immunostaining revealed the presence of proliferating endothelial cells, which indicated consequent angiogenesis, on the transcript level, only four genes out of 41 displayed significant changes. Moreover, the results appeared to be two-sided. Two pro-angiogenic genes; the receptor tyrosine kinase, Axl (2.01-fold), the hematopoietic growth factor, Il-3 (2.07-fold), and two anti-angiogenic genes; the soluble heat shock protein β-1 (Hspb1, 2.18-fold), interferon-γ inducible protein (Cxcl-10, 2.18-fold) were upregulated significantly.

### PACAP38 could Induce Microglia to Differentiate into M2 Phenotype via VPAC2 Receptor

**Fig. 6:**
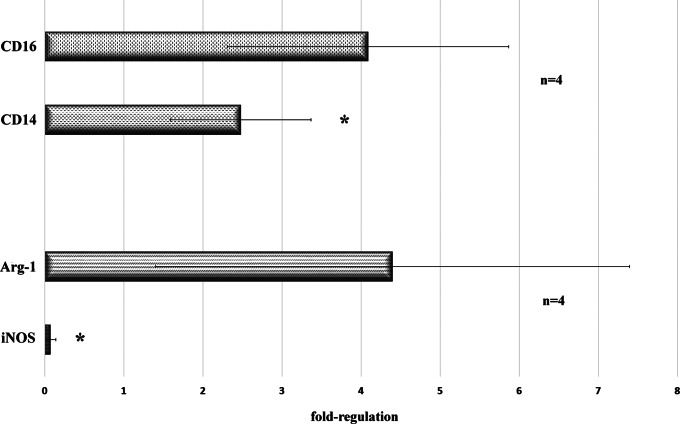
Gene expression ratios of M2 (CD16; Arg-1) and M1 (CD14; iNOS) microglia markers in 1-year-old PACAP38-treated retinas. Mean values ± SD of four biological replicates are given. Statistical comparisons were performed by one-way ANOVA analysis. Asterisks indicate p-values ≤ 0.05

Our previous analyses pointed to the fact that microglia were activated due to PACAP38 treatment. Since it is well-established that the microglia population is a complex and diverse assembly featured by numerous phenotype-related actions (Jurga et al., 2020), it was crucial to investigate, which phenotype is predominant in the PACAP38-treated retinas. Therefore, ratios of M1 and M2 markers were assessed in PACAP38-treated versus vehicle control samples. In Fig. 6, the expression of two key marker pairs can be compared in the PACAP38-treated retinas. Both CD16 and CD14 expressions were detectable, however, the CD16 message level was approximately 2-fold higher. In the case of the Arg-1/iNOS ratio, the tremendous difference detected is caused by the fact that 3 PACAP38-treated samples out of 4 lacked iNOS expression thus overall Arg-1 expression was approximately 60-fold higher in the retinas receiving PACAP38 treatments. The CD16^high^/CD14^low^ and Arg-1^high^/iNOS^low^ ratios indicate that although both M1 and M2 phenotypes are present, the microglia cells dominantly polarized into M2 state in response to PACAP38 injections.

**Fig. 7:**
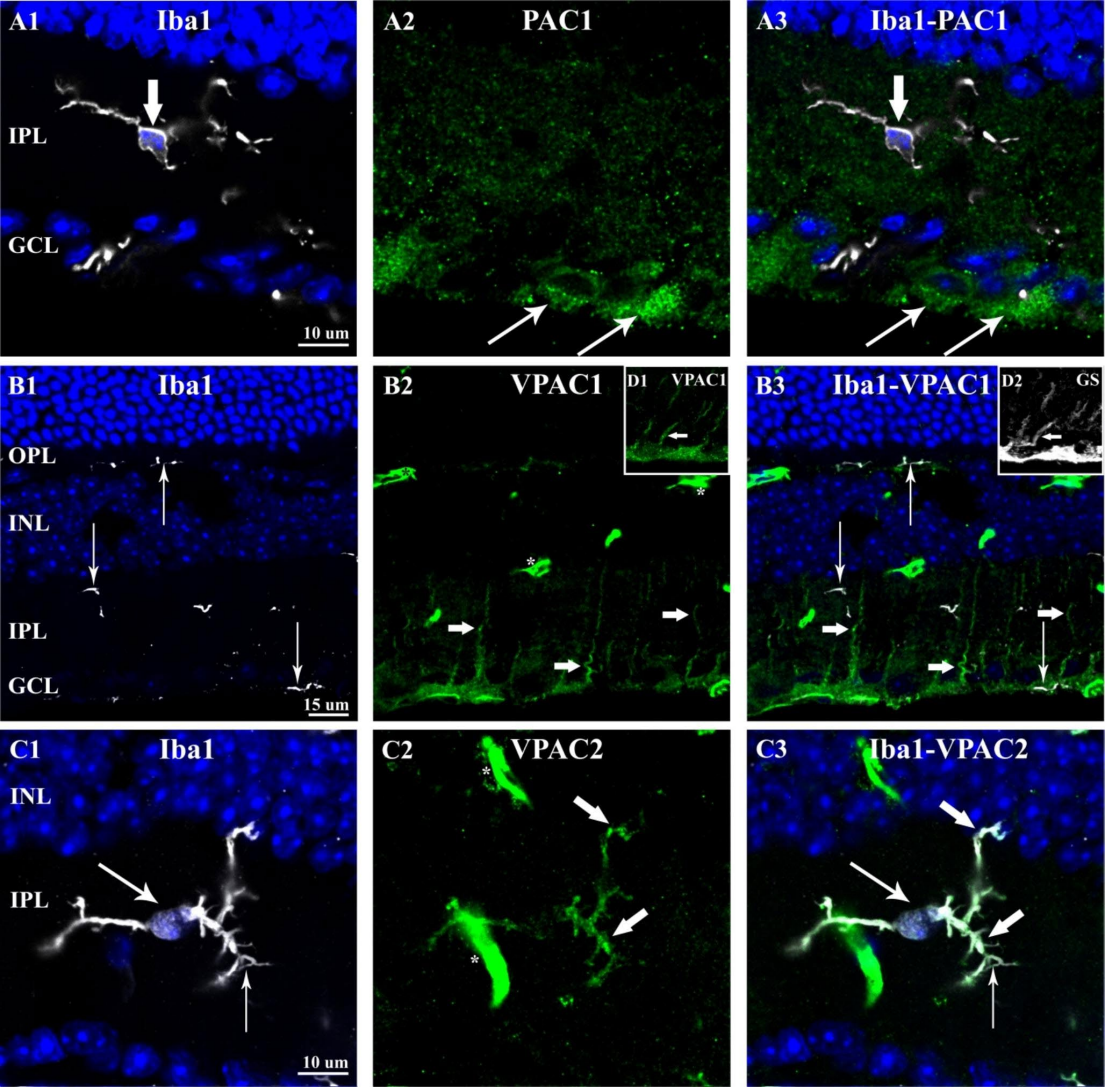
Co-localization between Iba-1 and PACAP38 receptors (PAC1, VPAC1, VPAC2) in the 1-year-old control retina. (A1) shows Iba-1-IR microglia (thick arrow). In the (A2) panel, PAC1-IR cells are observed only in the GCL (thin arrows). (A3) Merged pictures demonstrate that Iba-1 immunostaining does not co-localize with PAC1 immunostaining. In (B1), Iba-1-IR microglia processes are shown (thin arrows). (B2) VPAC1-IR processes (thick arrows) are seen in the IPL that do not co-localize with Iba-1 as demonstrated in (B3). (C1) An Iba-1-IR microglia somata and processes are marked with thin arrows. In (C2), VPAC2 immunostaining reveals the same cell with processes (thick arrows). (C3) shows a complete colocalization between Iba-1 and VPAC2 labelings. D1 and D2 seen as inserts in B2 and B3, respectively, demonstrate the colocalization between VPAC1-bearing processes and GS (white arrows). White asterisks in B2 and C2 mark non-specific staining in blood vessels due to mouse-on-mouse phenomenon (OPL-outer plexiform layer; INL-inner nuclear layer, IPL-inner plexiform layer; GCL-ganglion cell layer)

Undoubtedly, PACAP38 did have an impact on the retinal microglia population. Therefore, we examined next if microglial cells express PACAP receptors to see whether PACAP38 has a direct way to act on them. To address this issue, co-localization between Iba-1 and the three major PACAP38 receptor types (PAC1, VPAC1, VPAC2) was investigated. Figure 7A1-A3 show that Iba-1-IR microglia cell (A1, thick arrow) did not express PAC1-receptor, in contrast with the neurons of the GCL that were PAC1-positive (A2, thin arrows).

Similarly, Iba-1/VPAC1 immunolabeling revealed no overlapping (Fig. 7B1-B3). VPAC1-immunoreactivity was detected in thin processes spanning the IPL (Fig. 7B2, small arrows), which lacked Iba-1 signal. These processes appeared to be glutamine synthetase (GS)-positive (Fig. 7D1-D2) indicating that Müller-cells do express VPAC1. At last, we found that microglial cell somata, as well as processes (Fig. 7C1, thin arrows), expressed VPAC2-receptor (Fig. 7C2, thick arrows). For quantification purposes, 40 Iba-1-IR cells were examined sampled from both peripheral and central retinal sections equally. Seemingly, all retinal microglia cells were VPAC2 positive suggesting that PACAP38 could regulate the entire microglia population signaling through VPAC2 receptor.

## Discussion

Since its discovery [22], PACAP38 has emerged as a promising experimental therapeutic modality in retinal pathologies. A large set of scientific reports has been issued demonstrating that PACAP exerted neuroprotective effects in retinal disorders such as diabetic retinopathy, UV-A triggered apoptosis, ischemia, optic neuritis, traumatic optic neuropathy, and glaucoma due to its anti-apoptotic and/or anti-inflammatory action [23–28]. Nonetheless, most of the studies investigate the acute benefits of this multi-facetted neuropeptide and data on its multiple administration and long-term effects are scarce at best. In the present study we examined the most important biological sequelae of multiple and long-term PACAP38 administration in the context of cell proliferation, inflammatory status, and angiogenesis on the adult mouse retina.

### TH,-and SOM Amacrine Cells Express PAC1-receptor

Since we sought PACAP-receptor expression specifically in TH,- SOM, and microglia cells, PAC1,- VPAC1, and VPAC2-immunoreactivity were not studied systematically in this study. We show that both dopaminergic amacrine (Fig. 1A1-A3) and somatostatinergic cells (Fig. 1B1-B3; C1-C3) express PAC1 receptor thus demonstrating that PACAP38 may have an impact on their physiology including probably the cell cycle. We also observed numerous PAC1-expressing cells in the GCL and in the proximal INL. In a previous report, we have demonstrated that PAC1-IR cells in the rat INL and GCL, were mainly amacrine,- and horizontal cells but the majority of the ganglion cells were also labeled [10, 11]. PAC1-immunoreactivity in mouse thus seems to be very similar to that of the rat retina. However, the exact identities of these cells have yet to be characterized.

Robust expressions of VPAC1 and VPAC2 have been reported in the mouse retina through receptor autoradiography that did not allow the identification of the retinal cells [29]. Here, by using immunohistochemistry we described that Müller-glia (Fig. 7B1-B3) and microglial cells (Fig. 7C1-C3) may respond to PACAP38/VIP via VPAC1 and VPAC2 receptors, respectively. Unfortunately, experimental evidence is not available in the literature to interpret VPAC1-expression in Müller-cells. However, our data on VPAC2-expression in microglia will be discussed below.

***Proliferating cells of the PACAP38-treated retina include microglia, endothelial cells, and neurons of the GCL but not TH,-or SOM amacrine cells***.

We previously reported that intravitreal PACAP38 injection on the first postnatal week caused an increase of the PCNA level in 24 h and a concomitant increase of the horizontal cell number in rat retina [6]. The finding suggested that the mechanism of the elevated TH,-SOM cell numbers might be an induced cell division. However, by using a comprehensive molecular proliferation array, we found the puzzling Janus face of PACAP38. While changes of 17 cell cycling factors demonstrated the facilitating effect of PACAP38 on the 1-year-old retina (Table 4), alteration of 12 genes indicated that the long-term effect of PACAP38 could be the opposite (Table 5). As the latter was consistent with the anti-proliferative effects of PACAP38 in neonatal retinal explants [16, 30], the proliferative effects were analyzed in further detail next.

Visualization of the mitotic cells led to unexpected yet interesting results. A few PCNA-IR SOM cells were found exclusively in the vehicle control group (Fig. 2A1-A3) whereas none was seen in the PACAP38 treated retinas (Fig. 2B1-B3). Furthermore, we could not observe any PCNA-IR TH cells either in vehicle control or PACAP38-treated retina. Thus, the mitogenic activity, originally proposed to explain the increased cell number following PACAP38 treatments was not present in the cell populations of our interest. However, it is important to point out that the retinas were examined 3 months post-injection. Therefore, induction of TH or SOM-cells to re-enter the cell cycle cannot be ruled out by the present study. A different workflow designed on a more refined time scale will clarify this issue. In addition, progenitor activation or transdifferentiation as potential underlying mechanisms must be taken into consideration in future investigations as well.

Contrary to the TH and SOM positive amacrine population, we did find some PCNA positive cells, exclusively in the GCL of the PACAP38-treated retinas (Fig. 2C1-C3; D1-D3). The characteristic morphology and lack of Iba1-immunoreactivity (microglia) proved that PACAP38 induced neurons to divide, however, lack of the TdTomato signal excluded SOM-cells. The exact identification of these cells, whether they are ganglion or displaced amacrine cells will be the subject of future studies.

Furthermore, numerous proliferating endothelial cells were also spotted in the treated retinas (Fig. 3D; E1-E4). The phenomenon is discussed in detail in the last section.

The most abundant PCNA-positive cells in both controls and PACAP38-treated retinas appeared to be microglia cells (Fig. 3A1-A3; B1-B3, C1-C3). Therefore, instead of the original hypothesis to explain the PACAP38-induced increased amacrine cell numbers, our focus shifted toward the investigation of retinal microglia.

#### Chronic PACAP38-treatment Stimulates Retinal Microglia to Polarize into M2-phenotype

Beside their significant functions in ocular homeostasis, retinal microglia has been reported to have pivotal roles in the progression of retinal pathologies. Their implication in the leading causes of blindness including glaucoma, diabetic retinopathy, even the inherited retinitis pigmentosa, just to mention a few, confers an outstanding therapeutic value on these resident macrophages [31–33]. Strategies targeting microglia include depletion, reprograming, and blockage of downstream effectors [1].

As it has been mentioned in the first paragraph, the anti-inflammatory effect of PACAP38 also received great research attention including its interactions with microglial cells. Inhibition exerted by PACAP38 is manifested by reduced production of pro-inflammatory cytokines, tumor necrosis factor α, or NO, also decreased expression of NADPH oxidase [34–36]. In the retina, PACAP38 caused a significant proliferation of the microglia 3 days post-injection [37]. In our study, three months post-injection, the microglial number did not differ significantly between the vehicle control and PACAP38-treated tissues (Fig. 4) despite the increased transcription of Csf1- Csf1r genes (Fig. 5). At the same time, the area they occupied was significantly smaller in the PACAP38-treated retinas compared to the vehicle control. The unaltered microglial cell number led to the conclusion that in 3 months after the injection, the proliferation of microglial cells is not facilitated. However, the reduced area fraction suggested an activation, which was confirmed by the gene expression data. Gene expression data obtained from the neuroinflammation panel (Fig. 5) suggest a diverse functional state of the retinal microglia population that might include an enhanced migration (Mmp12, Cxcl10) synaptic remodeling (C3), neuron-microglia communication, which maintains non-neurotoxic state (Cx3cr1), phagocytosis (Tlr2, Trem2) in the PACAP38 treated retina. On the other hand, a set of inflammatory regulators (Tnf, Il-1b, Nlrp3, P2rx7) were also upregulated [1, 38–40].

A large cohort of molecules is used to define microglial profiles, which vary from neutral to pro-inflammatory (M1) or anti-inflammatory (M2) [38, 41]. Seemingly, in the PACAP38-treated retina, the microglia population is featured by mixed phenotypes. On one hand, the presence of non-activated (homeostatic) microglia [42] is indicated by the upregulated Cxcr1, Csf1r, Ctss, and Tmem119. The increased expression of markers such as Tnf, Cd86, Mmp12, and Ccl5 indicate M1 microglia activation. Nevertheless, significant elevation of Il1rn indicated the presence of non-inflammatory M2 microglia [43] thus we examined four genes in pairs, Arg1/iNOS and CD16/CD14 to establish the dominant microglia phenotype in the PACAP38-treated retinas. Despite the upregulation of the M1-related markers, the Arg1^high^/iNOS^low^, also the CD16^high^/CD14^low^ ratios clearly indicate that the microglia cells were triggered to differentiate dominantly into tissue-supportive M2 phenotype. Our finding is in congruence with the report of Brifault and co-workers, who revealed a PACAP38-induced M2 microglia polarization following strokes [44]. In respect of the retina, data mining led to only one study. Wada and colleagues [37] investigated the short-term effect of a single PACAP injection on retinal microglia in N-methyl-D-aspartic acid (NMDA)-treated retina. They found that PACAP38 increased the microglia number, however, did not change the expression of either M1 markers (TNFα, interleukin 6) or M2 markers (Il-4, Chi3L3) but promoted acquired deactivation of the microglia cells. Comparatively, the long-term effect of repeated PACAP38 injections examined in the current study caused both M1 and M2 polarization of the retinal microglial population and with the surprising finding of Yang et al. [35] that PACAP38 was unable to exert neuroprotection in the absence of microglia. Based on an interesting and recent discovery, which also supports our results microglial cells could be consider as allies providing trophic support and even mediating cell survival in retinal degenerative diseases [45, 46].

Furthermore, we revealed for the first time that the aforementioned effects were mediated exclusively via VPAC2 receptor in the murine retina, another interesting finding to discuss. In view of the immune system, VPAC1 is considered to be the key receptor, which mainly mediates PACAP38/VIP actions. VPAC2 has also been implicated in numerous actions whereas PAC1 receptor has been reported to be rather underrepresented in immune cells. Regardless of their abundance, all types of PACAP receptors evidently induce a tolerogenic or anti-inflammatory profile of the immune cells [47]. A compelling amount of data have been published that PACAP38 affects microglia through VPAC1 and PAC1 receptors for VPAC2 expression are absent in microglial cells in the central nervous system (e.g. spinal cord, cerebral cortex, hippocampus) [34, 48, 49]. In contrast, the immortalized BV-2 microglial cell line does express PAC1, VPAC1, and VPAC2 receptors [36, 50]. According to our findings, retinal microglia exhibits only VPAC2 receptor in the mouse retina and VPAC1 and PAC1 receptors are expressed in Müller-glia and in retinal neurons, respectively.

#### PACAP38-induced Microglia Activation might Enhance Angiogenesis in the Adult Retina

Angiogenesis, the formation of new vessels from pre-existing capillaries is an essential process in all tissues, especially under hypoxic conditions. Nonetheless, when it becomes imbalanced, angiogenesis can cause severe visual impairment described in various retinal diseases, such as diabetic retinopathy, age-related macular degeneration, and retinal vessel occlusion [51]. The role of PACAP38 in angiogenesis has been meticulously studied under pathological conditions. Accordingly, the peptide was proved to inhibit both vascular endothelial growth factor (VEGF) expression and pathway [52, 53], suppress the proliferation of the endothelial cells [54], and, at last but not least, prevented neovascularization of retinal pigmented epithelial cells [55]. Our results, the upregulation of Hspb-1 confirmed these reports as Hspb-1 produced by endothelial cells antagonizes VEGF signaling [56]. In addition, Cxcl-10, the pleiotropic member of the chemokine family (also known as IP-10), was also reported as an anti-angiogenic factor [57]. However, unexpectedly enough, numerous proliferating endothelial cells were spotted in the PACAP38-injected retinas. In concordance, the upregulation of two key regulators in angiogenesis was measured: Axl, Il-3. Axl is a receptor tyrosine kinase, which contributes to VEGF signaling. As a powerful angiogenic mediator, Axl has been widely studied in retinal as well as tumor angiogenesis [58, 59]. Il-3 is another well-known regulator of both vasculogenesis and angiogenesis, which stimulates VEGF gene transcription. Il-3 could be released from activated T-cells, astrocytes or even retinal ganglion cells [60–63]. In addition, the message level of matrix metalloproteinase 14 (MMP14) was also significantly increased by PACAP38. Although MMP14 is not considered a classical angiogenetic factor, it was demonstrated to have a great impact on neovascularization along with other microglia-related molecules [64]. Hence, the assumed angiogenesis could be very well the consequence of the parallel microglia activation, which moves the balance in favor of the angiogenic factors.

In sum, our results demonstrate promise indicating that PACAP38 could contribute to the alleviation of inflammatory retinal diseases by reprogramming microglia into a preferable M2 phenotype. However, the development of new blood vessels as an unwanted consequence of chronic PACAP38 treatment has to be taken into account.

## Data Availability

All associated data is available upon request from the corresponding author.
